# Supplementary material for: The impact of within-vector parasite development on the extrinsic incubation period
Source: R Soc Open Sci. 2020 Oct 7;7(10):192173. doi: 10.1098/rsos.192173 (PMC7657899; doi:10.1098/rsos.192173)
Supplement: Supplementary Material [file rsos192173supp1.pdf]

rsos.royalsocietypublishing.org R. Soc. open sci. 000000

Supplementary Material for

The impact of within-vector parasite development on the extrinsic incubation period

Lauren M. Childs and Olivia F. Prosper

Sporozoite score versus sporozoite number

In Dawes [16], the sporozoite abundance is reported as a score between 0 and 4, rather than a number. Our model framework tracks the number of sporozoites and, thus, we must transform the sporozoite score from the data to a sporozoite number (or the sporozoite number from the model to sporozoite score) for comparison. In this work, we use the reported score as a power of 10. This gives a maximum of sporozoites present given a particular score. We also considered the two other ways to scale from the sporozoite score listed in Table 2 of the Dawes thesis: (i) arithmetic and (ii) "pseudo"-geometric mean. Although listed as geometric, it is not truly a geometric mean; hence, we refer to this version as "pseudo-geometric." As the average scores are not whole number, we describe how we use these methods. For (i) arithmetic, we take the arithmetic mean from $10^{\text{floor(average score)}}$ to round($10^{\text{(average score)}}$). Thus, if the average score was 1.8, then we would consider the arithmetic mean of the numbers from 10 to 63 or a value of 36.5. For (ii) pseudo-geometric, we raised 10 to the arithmetic mean of the score and the floor(score). Thus, if the average score was 1.8, then we would consider 10 raised to the average value of 1 and 1.8 or $10^{1.4}$, a value of 25.1. Using these procedures, we ran a 10 set multistart (i.e. fitting with 10 different starting parameter sets) for each of N =2, 3, 10, 20, 30, 40, 50, 75, and 100 for Model 1 as well as a 10 set multistart run for Model 2. These are the same choices for multistart used elsewhere.

Our results were qualitatively quite similar under the three scenarios. In particular, the EIP values did not differ appreciably based on the metric chosen to transform the sporozoites scores (Figure S2). As the average score falls between whole number scores, it is impossible to know if the arithmetic and pseudo-geometric mean calculations are over or under-estimating the true values. While the 10 raised to the average score will necessarily overestimate the value, and thus we choose this metric for our results.

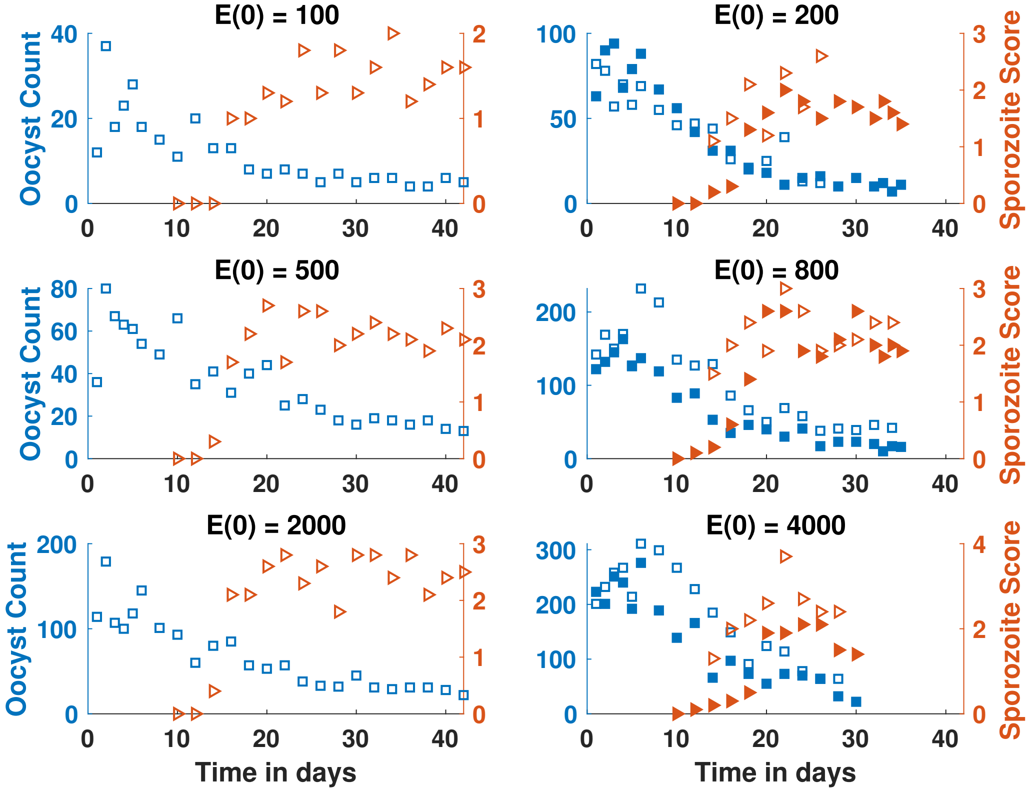

Figure S1: **Oocyst count and sporozoite score data.** Data are extracted from [16]. Oocyst count is represented by squares, and sporozoite score is represented by triangles. Filled markers denote replicate experiments that were performed for initial ookinete numbers of $E(0) \in \{200, 800, 4000\}$.

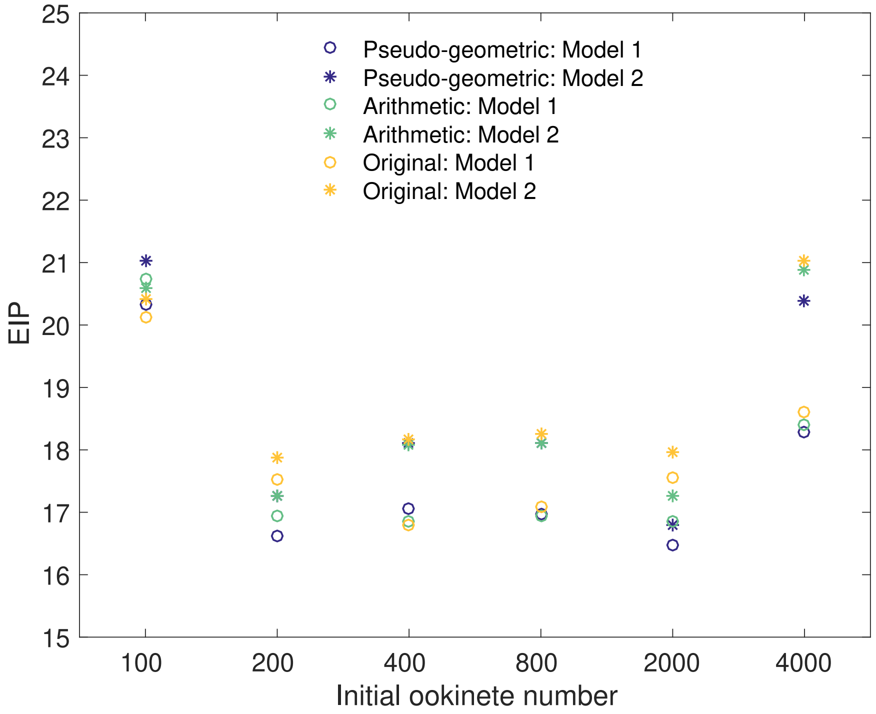

Figure S2: **EIP with different sporozoite score transformations.** Three methods are used to transform the sporzoite score to abundance: pseudo-geometric (blue), arithmetic (green) and anti-log (orange), which was used in all results in the main text. Results from Model 1 are shown with open circles and from Model 2 with stars.

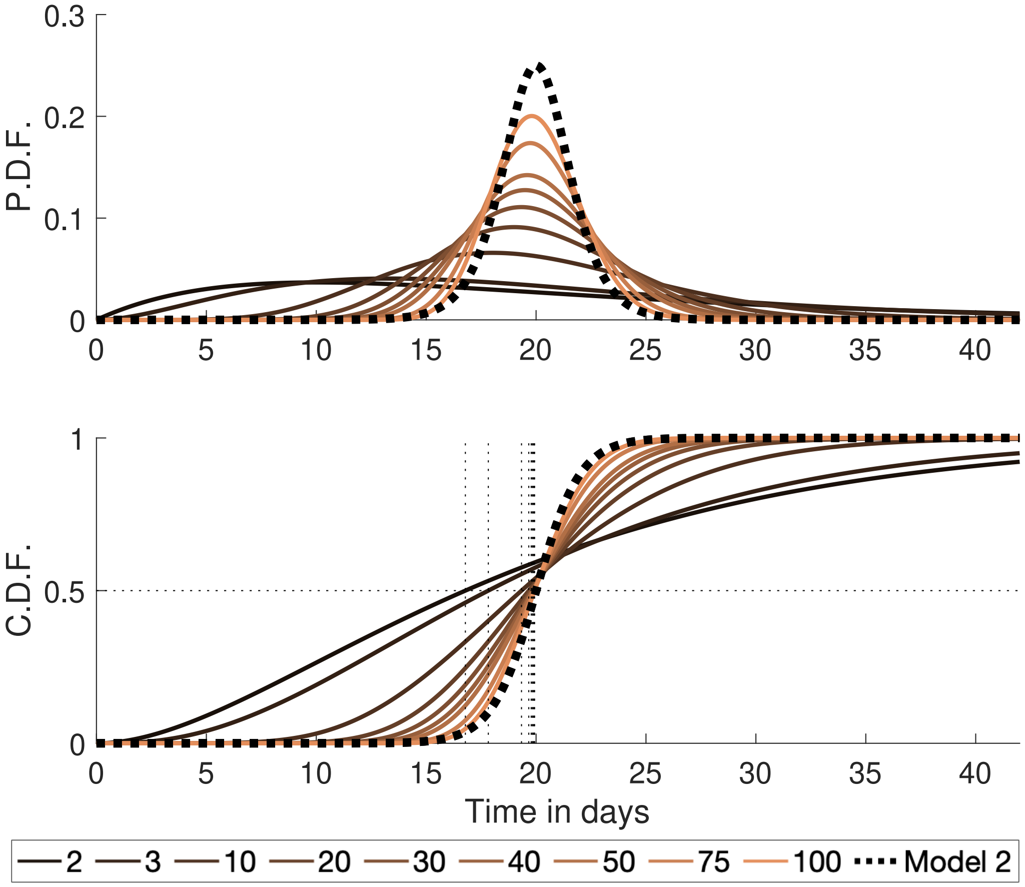

Figure S3: **Probability density function (PDF) and Cumulative distribution function (CDF) of gamma-distributed bursting versus time-dependent bursting.** Solid lines correspond to Model 1 with gamma-distributed bursting corresponding to shape parameter N (number of bursting oocyst stages) and scale $20/N$, $N \in \{2, 3, 10, 20, 30, 40, 50, 75, 100\}$. The black dashed line corresponds to Model 2 with time-dependent bursting. Note that for Model 2, the time-dependent rupture function is not a true cumulative distribution function, and that its corresponding 'pseudo-PDF' is a numerical approximation of its derivative. Vertical dotted lines represent the times at which the CDF equals 0.5 for Model 1, for each N.

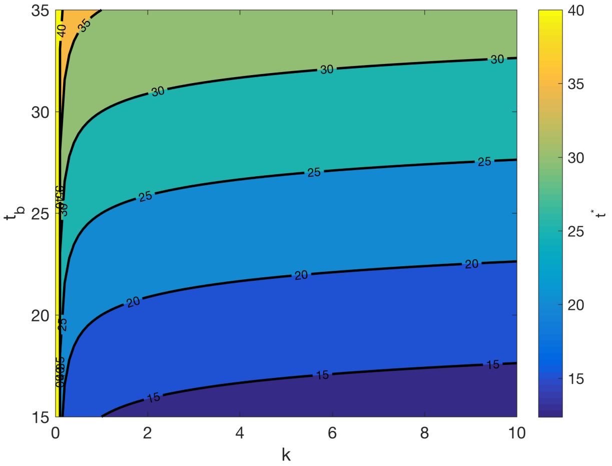

Figure S4: **Dependence of t^* on k and t_b.** Lower t^* in blue to higher t^* in yellow. Relationship of t^*, k, and t_b from the simplified model found in Equation 4.1.

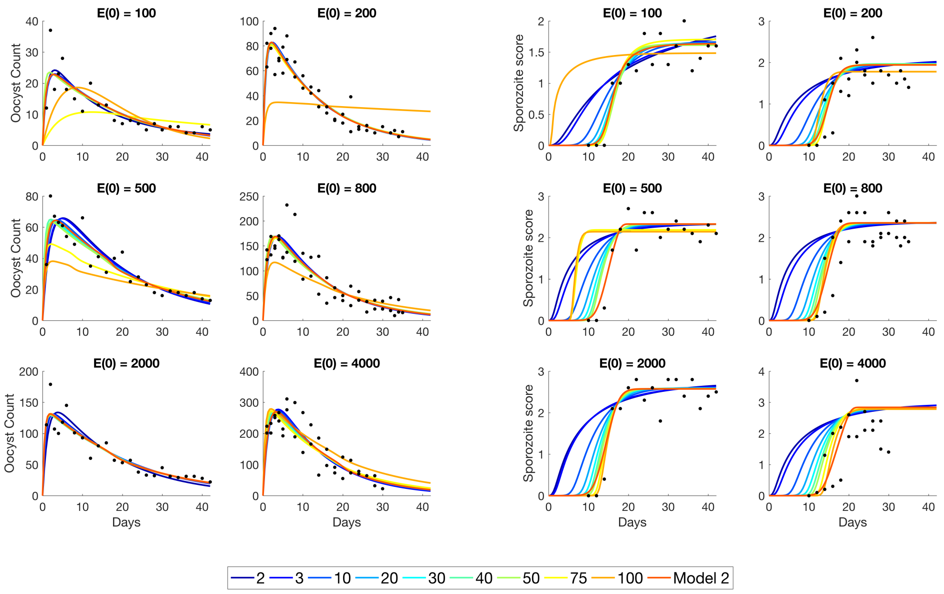

Figure S5: **Best fit model solutions from multistart.** Best fit to oocyst count and sporozoite score for Model 1 ($N > 1$) and Model 2 using multistart. Colored lines represent the indicated Model fit and dots represent data. The left six panels are oocyst count at varying initial ookinete numbers while the right six are sporozoite score. Panels are ordered by initial ookinete number, as indicated by the titles.

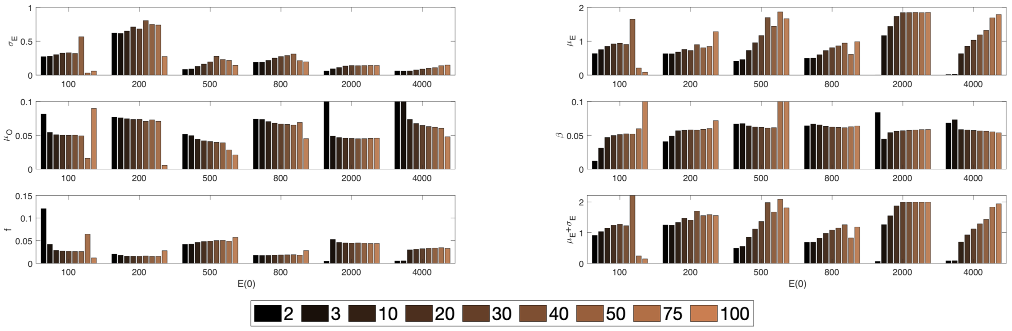

Figure S6: **Optimized parameter estimates for Model 1**. Parameter estimates using multistart for each initial ookinete density $E(0)$ across different numbers of rupturing oocyst stages $N \in \{2, 3, 10, 20, 30, 40, 50, 75, 100\}$. Note: the y-axis limits have been truncated for μ_O and β to highlight that when the model fits the data reasonably well, i.e. lower AICc, the estimates of parameters μ_O, β and f are similar across N.

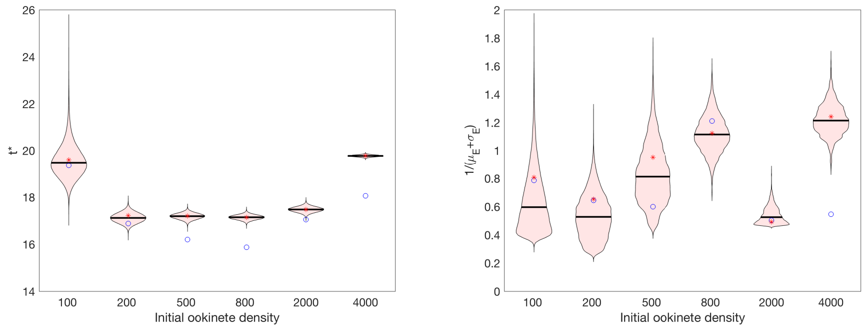

Figure S7: **Variation in contributions to EIP.** The median time to bursting as an oocyst, t^* (left), and the time spent as an ookinete $1/(\mu_E + \sigma_E)$ (right). In all plots, the violin plot shows the variation in the MCMC fitting with the black bars representing the median. Red stars are the values from Model 2 fitted with multistart and blue circles are the values from Model 1 fitted with multistart. All optimized parameters are found in in Table 2 with 95% highest density posterior intervals found in Table S4.

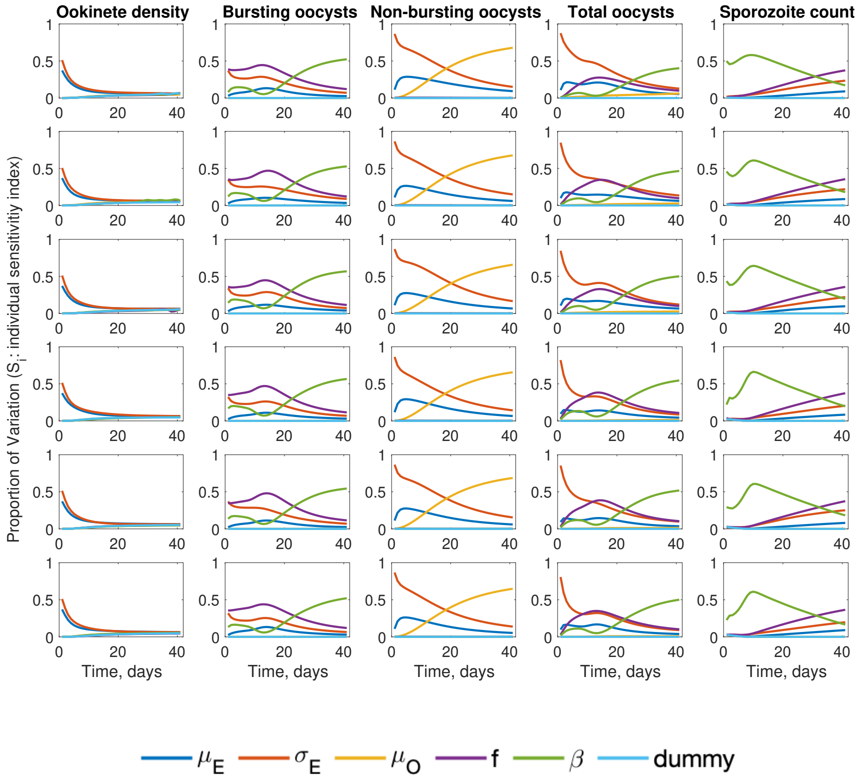

Figure S8: **Global individual sensitivity of Model 1 output over time.** The individual sensitivity index, S_i, for each parameter. Outputs include Model 1 compartments and the total oocysts, in order of columns: ookinete, E; bursting oocysts, $O = \sum_i^N O_i$; non-bursting oocysts, O_d; total oocysts, $O + O_d$; and sporozoites, S. Rows are increasing initial ookinete number: $E(0) \in \{100, 200, 500, 800, 2000, 4000\}$. For each $E(0)$ the optimal N, shown in Table 2, is used.

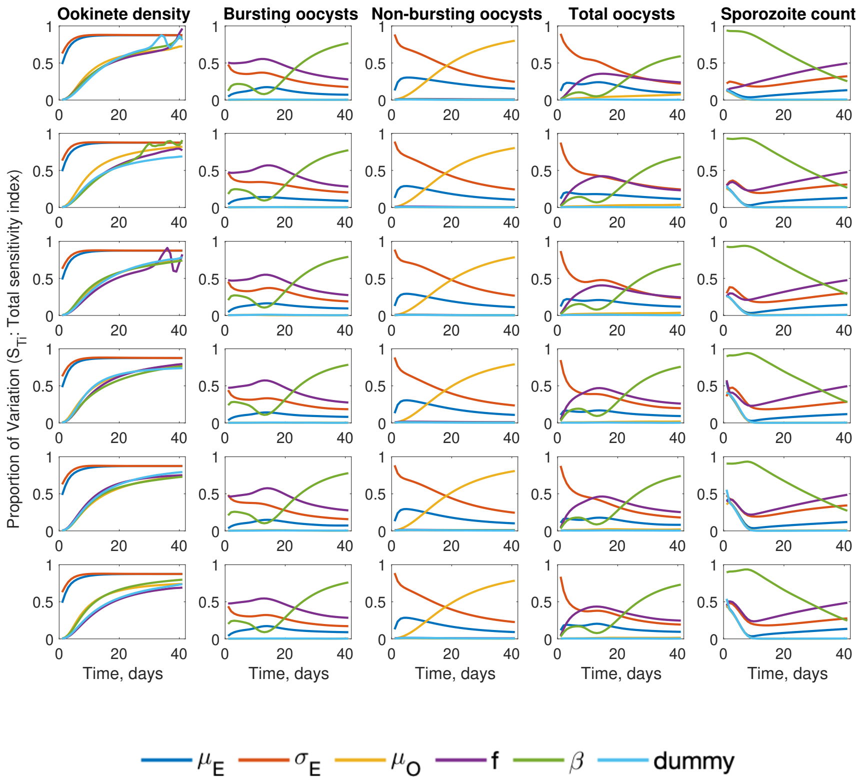

Figure S9: **Global total sensitivity of Model 1 output over time.** The total sensitivity index, S_{Ti}, for each parameter. Outputs include Model 1 compartments and the total oocysts, in order of columns: ookinete, E; bursting oocysts, $O = \sum_i^N O_i$; non-bursting oocysts, O_d; total oocysts, $O + O_d$; and sporozoites, S. Rows are increasing initial ookinete number: $E(0) \in \{100, 200, 500, 800, 2000, 4000\}$. For each $E(0)$ the optimal N, shown in Table 2, is used.

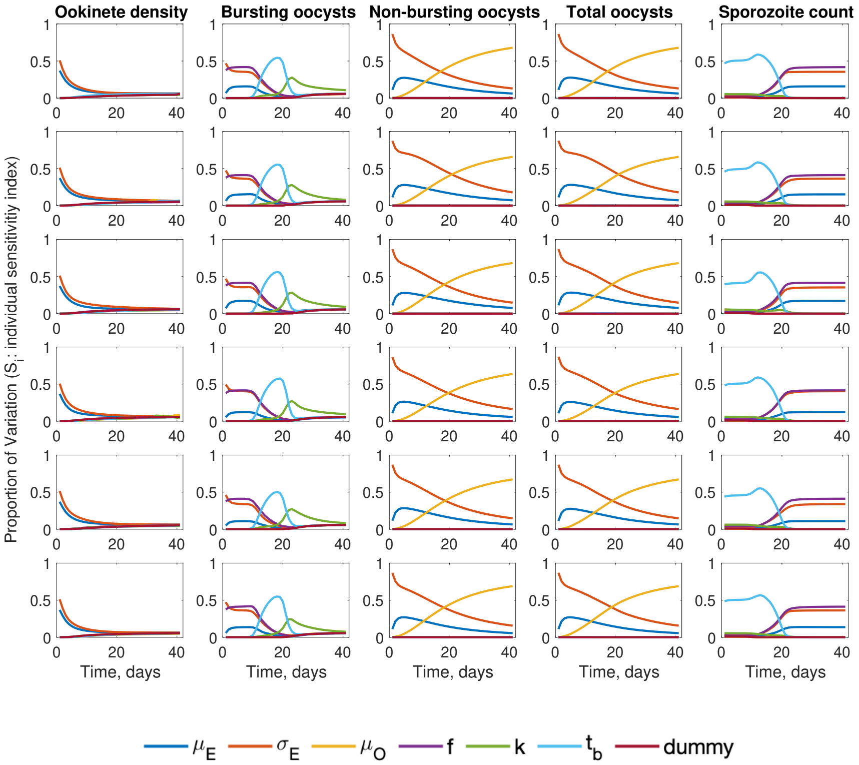

Figure S10: **Global individual sensitivity of Model 2 output over time.** The individual sensitivity index, S_i, for each parameter. Outputs include each Model 2 compartment and the total oocysts, in order of columns: ookinete, E; bursting oocyst, O; non-bursting oocyst, O_d; total oocysts, $O + O_d$; and sporozoites, S. Rows are increasing initial ookinete number: $E(0) \in \{100, 200, 500, 800, 2000, 4000\}$. For each $E(0)$ the optimal N, shown in Table 2, is used.

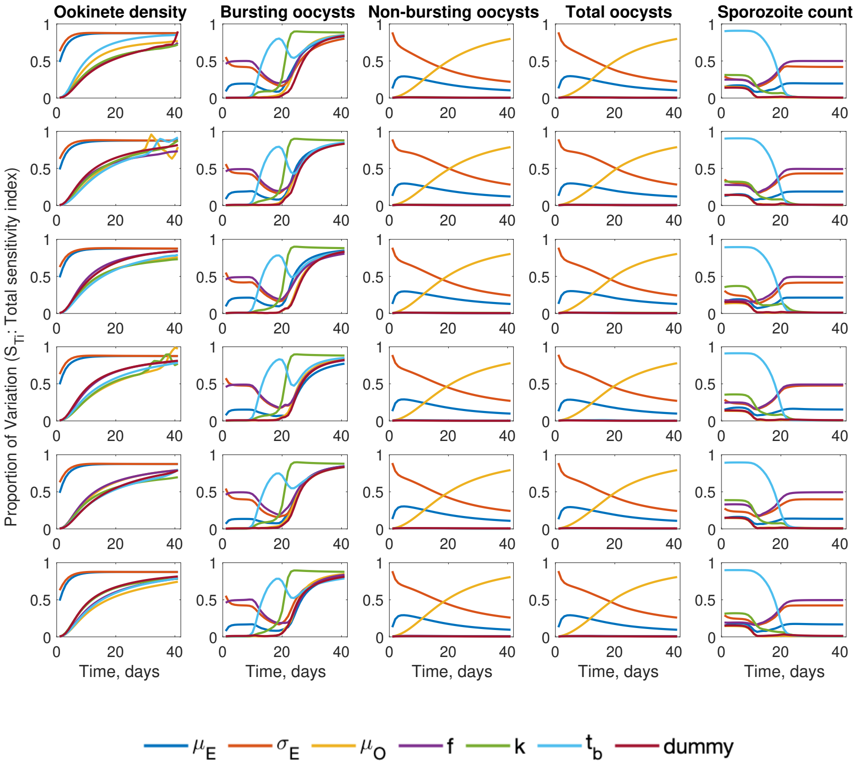

Figure S11: **Global total sensitivity of Model 2 output over time.** The total sensitivity index, S_{Ti}, for each parameter. Outputs include each Model 2 compartment and the total oocysts, in order of columns: ookinete, E; bursting oocyst, O; non-bursting oocyst, O_d; total oocysts, $O+O_d$; and sporozoites, S. Rows are increasing initial ookinete number: $E(0) \in \{100, 200, 500, 800, 2000, 4000\}$. For each $E(0)$ the optimal N, shown in Table 2, is used.

	Model 1									
$E(0)$	2	3	10	20	30	40	50	75	100	Model 2
100	10	9	8	10	10	10	10	10	10	10
200	10	10	10	10	10	10	10	10	10	9
500	10	10	9	10	10	10	10	10	10	9
800	10	10	10	10	10	10	10	10	10	10
2000	10	10	10	9	10	10	10	10	10	10
4000	10	9	10	10	9	10	10	10	10	10

Table S1: **Multistart convergence metrics.** The number of simulations out of ten multistart runs that converged, where convergence was determined by an exit flag of 1 or 2 and an objective function value less than 10^9 (the value returned by the objective function if the negative log-likelihood is undefined for a given parameter set). All data is available at the GitHub Repository. For Model 1, the column headers indicate the number of oocyst sub-compartments, N.

E(0)=100	Point est.	Upper C.I.
σ_E	1.00	1.01
μ_E	1.00	1.01
f	1.00	1.00
t_b	1.00	1.00
k	1.00	1.01
μ_O	1.00	1.00
E(0)=200	Point est.	Upper C.I.
σ_E	1.02	1.05
μ_E	1.02	1.05
f	1.01	1.02
t_b	1.01	1.02
k	1.01	1.02
μ_O	1.00	1.01
E(0)=500	Point est.	Upper C.I.
σ_E	1.05	1.10
μ_E	1.05	1.10
f	1.01	1.01
t_b	1.01	1.02
k	1.01	1.02
μ_O	1.00	1.01
E(0) = 800	Point est.	Upper C.I.
σ_E	1.01	1.02
μ_E	1.01	1.02
f	1.01	1.02
t_b	1.01	1.02
k	1.02	1.03
μ_O	1.01	1.02
E(0)=2000	Point est.	Upper C.I.
σ_E	1.01	1.03
μ_E	1.01	1.03
f	1.00	1.00
t_b	1.01	1.02
k	1.02	1.03
μ_O	1.00	1.00
E(0)=4000	Point est.	Upper C.I.
σ_E	1.02	1.05
μ_E	1.02	1.06
f	1.01	1.02
t_b	1.00	1.00
k	1.00	1.00
μ_O	1.01	1.02

Table S2: **Gelman-Rubin convergence diagnostic for each initial ookinete number.** Convergence computed with the gelman.diag function in R. For each initial condition, five chains of at least 100,000 steps are combined. The chains and additional details are available at the GitHub Repository.

	Model 1									
E(0)	2	3	10	20	30	40	50	75	100	Model 2
100	437	424	381	366	363*	364	368	544	637	370
200	1921	1877	1717	1633	1602	1592	1586*	1588	2463	1590
500	2110	2028	1733	1568	1489	1444	1411	2560	2712	1309*
800	5333	5224	4800	4532	4396	4318	4271	4237	4503	4194*
2000	2749	2654	2210	2013	1929	1883	1857	1832*	1833	1853
4000	22658	22478	21359	20530	20037	19707	19474	19130	19182	18268*

Table S3: **AICc values for the optimal multistart parameter set.** Column headers for Model 1, give the number of oocyst sub-compartments, N. Visual representation in Figure 2. A * indicates the model with the lowest AICc for a given initial ookinete number.

$E(0) =$ 100	lower	upper	lower	upper	lower	upper	lower	upper	lower	upper
σ_E	0.205	0.668	0.208	0.705	0.207	0.699	0.219	0.676	0.205	0.668
μ_E	0.684	1.999	0.698	2.000	0.658	1.996	0.699	2.000	0.684	1.999
f	0.022	0.034	0.022	0.034	0.022	0.034	0.022	0.034	0.022	0.034
t_b	14.955	18.002	15.043	17.890	15.045	18.057	15.021	18.027	14.955	18.002
k	0.093	0.456	0.105	0.436	0.102	0.460	0.100	0.454	0.093	0.456
μ_O	0.038	0.061	0.038	0.060	0.039	0.061	0.038	0.061	0.038	0.061
$E(0) =$ 200	lower	upper	lower	upper	lower	upper	lower	upper	lower	upper
σ_E	0.533	1.575	0.543	1.580	0.523	1.548	0.541	1.548	0.533	1.575
μ_E	0.556	1.831	0.549	1.807	0.516	1.760	0.526	1.764	0.556	1.831
f	0.013	0.017	0.014	0.017	0.013	0.016	0.013	0.016	0.013	0.017
t_b	16.120	19.459	16.018	19.374	16.138	19.619	16.159	19.582	16.120	19.459
k	0.391	7.887	0.362	7.510	0.374	9.000	0.403	8.853	0.391	7.887
μ_O	0.065	0.079	0.065	0.079	0.065	0.079	0.065	0.079	0.065	0.079
$E(0) =$ 500	lower	upper	lower	upper	lower	upper	lower	upper	lower	upper
σ_E	0.108	0.295	0.113	0.276	0.108	0.261	0.107	0.259	0.108	0.295
μ_E	0.606	1.812	0.628	1.708	0.653	1.625	0.587	1.568	0.606	1.812
f	0.043	0.055	0.043	0.055	0.043	0.055	0.043	0.055	0.043	0.055
t_b	18.609	19.981	18.715	19.995	18.749	19.970	18.681	20.002	18.609	19.981
k	3.534	9.997	3.783	10.000	3.923	10.000	3.736	10.000	3.534	9.997
μ_O	0.034	0.048	0.035	0.048	0.035	0.048	0.035	0.048	0.034	0.048
$E(0) =$ 800	lower	upper	lower	upper	lower	upper	lower	upper	lower	upper
σ_E	0.196	0.283	0.189	0.297	0.196	0.296	0.193	0.287	0.196	0.283
μ_E	0.525	0.830	0.502	0.878	0.528	0.878	0.520	0.853	0.525	0.830
f	0.017	0.019	0.017	0.019	0.017	0.019	0.017	0.019	0.017	0.019
t_b	17.225	18.770	17.241	18.717	17.311	18.648	17.210	18.839	17.225	18.770
k	1.018	3.504	1.079	3.490	1.089	3.251	1.008	3.711	1.018	3.504
μ_O	0.065	0.075	0.064	0.074	0.064	0.074	0.064	0.074	0.065	0.075
$E(0) =$ 2000	lower	upper	lower	upper	lower	upper	lower	upper	lower	upper
σ_E	0.107	0.161	0.108	0.161	0.113	0.159	0.111	0.160	0.107	0.161
μ_E	1.401	2.000	1.408	2.000	1.482	2.000	1.451	2.000	1.401	2.000
f	0.040	0.047	0.040	0.047	0.040	0.047	0.040	0.047	0.040	0.047
t_b	16.764	17.981	16.735	18.124	16.722	18.166	16.759	18.046	16.764	17.981
k	0.598	1.352	0.594	1.519	0.568	1.531	0.591	1.418	0.598	1.352
μ_O	0.041	0.050	0.041	0.051	0.041	0.050	0.041	0.050	0.041	0.050
$E(0) =$ 4000	lower	upper	lower	upper	lower	upper	lower	upper	lower	upper
σ_E	0.059	0.081	0.057	0.078	0.059	0.080	0.059	0.082	0.059	0.081
μ_E	0.605	0.912	0.587	0.878	0.625	0.897	0.604	0.929	0.605	0.912
f	0.032	0.036	0.032	0.036	0.032	0.036	0.032	0.036	0.032	0.036
t_b	22.240	22.486	22.227	22.477	22.226	22.471	22.238	22.484	22.239	22.486
k	8.758	10.000	8.696	10.000	8.729	10.000	8.743	10.000	8.757	10.000
μ_O	0.065	0.075	0.065	0.076	0.065	0.075	0.064	0.075	0.065	0.075

Table S4: **95 % Highest density posterior interval for parameters.** Calculated using HPDinterval in R with chains combined. Columns represent each of five MCMC chains.

$E(0)$	Chain	t_b, k	f, μ_O	σ_E, μ_E	f,σ_E	f,μ_E	μ_O, σ_E	μ_O, μ_E
	1	0.50	-0.58	0.91	-0.08	0.23	0.01	-0.29
	2	0.76	-0.59	0.91	-0.14	0.18	0.06	-0.24
100	3	0.47	-0.59	0.92	-0.10	0.21	0.03	-0.26
	4	0.74	-0.60	0.91	-0.04	0.29	-0.01	-0.32
	5	0.74	-.0.58	0.92	-0.07	0.23	-0.01	-0.29
	1	0.92	-0.58	0.97	0.17	0.33	-0.32	-0.46
	2	0.92	-0.59	0.97	0.14	0.31	-0.32	-0.47
200	3	0.94	-0.59	0.97	0.21	0.37	-0.30	-0.46
	4	0.93	-0.58	0.97	0.19	0.36	-0.33	-0.48
	5	0.90	-0.60	0.97	0.21	0.38	-0.34	-0.49
	1	0.93	-0.76	0.98	0.24	0.42	-0.30	-0.45
	2	0.91	-0.76	0.97	0.19	0.41	-0.23	-0.42
500	3	0.91	-0.76	0.96	0.17	0.42	-0.22	-0.42
	4	0.92	-0.75	0.95	0.16	0.43	-0.20	-0.42
	5	0.92	-0.77	0.96	0.14	0.39	-0.20	-0.40
	1	0.91	-0.75	0.95	0.38	0.61	-0.47	-0.66
	2	0.90	-0.75	0.96	0.42	0.61	-0.50	-0.66
800	3	0.92	-0.74	0.95	0.35	0.58	-0.45	-0.64
	4	0.90	-0.75	0.95	0.39	0.61	-0.48	-0.67
	5	0.91	-0.74	0.96	0.40	0.60	-0.49	-0.66
	1	0.91	-0.71	0.93	-0.14	0.20	0.06	-0.22
	2	0.92	-0.71	0.92	-0.10	0.26	0.04	-0.25
2000	3	0.89	-0.70	0.90	-0.24	0.17	0.15	-0.18
	4	0.92	-0.71	0.91	-0.25	0.15	0.18	-0.15
	5	0.90	-0.70	0.93	-0.16	0.20	0.07	-0.21
	1	0.77	-0.80	0.96	0.47	0.66	-0.53	-0.70
	2	0.79	-0.77	0.96	0.43	0.64	-0.50	-0.68
4000	3	0.78	-0.79	0.96	0.45	0.65	-0.52	-0.69
	4	0.76	-0.81	0.97	0.52	0.70	-0.58	-0.73
	5	0.79	-0.82	0.97	0.55	0.71	-0.60	-0.74

Table S5: **Parameter correlations in MCMC chains.** Correlation coefficient pairwise between parameters. All correlation coefficients including visual representation can be found in the GitHub Repository.
